# The first limb-sparing use of histotripsy for canine osteosarcoma

**DOI:** 10.1038/s41598-026-42319-z

**Published:** 2026-03-23

**Authors:** Elliana R. Vickers, Lauren N. Ruger, Alayna N. Hay, Ny T. C. Luong, John S. Kett, Summer Vander Kooi, Sheryl L. Coutermarsh-Ott, Gregory B. Daniel, Timothy J. Ziemlewicz, Steven B. Soliman, Gunjan B. Malhotra, Adam D. Maxwell, Eli Vlaisavljevich, Joanne Tuohy

**Affiliations:** 1 Department of Biomedical Engineering and Mechanics, Virginia Polytechnic Institute and State University, Blacksburg, VA USA; 2 Virginia Tech Animal Cancer Care and Research Center, Virginia-Maryland College of Veterinary Medicine, Roanoke, VA USA; 3 Doctor of Veterinary Medicine Program, Virginia-Maryland College of Veterinary Medicine, Blacksburg, VA USA; 4 School of Animal Sciences, Virginia Polytechnic Institute and State University, Blacksburg, VA USA; 5 Department of Biomedical Sciences and Pathobiology, Virginia Polytechnic Institute and State University, Blacksburg, VA USA; 6 Department of Small Animal Clinical Sciences, Virginia-Maryland College of Veterinary Medicine, Blacksburg, VA USA; 7 Department of Radiology, University of Wisconsin, Madison, WI USA; 8 Division of Musculoskeletal Radiology, Department of Radiology, University of Michigan/Michigan Medicine, Ann Arbor, MI USA

**Keywords:** Osteosarcoma, Canine, Focused ultrasound, Fractionated, Histotripsy, Ablation, Cancer, Diseases, Medical research, Oncology

## Abstract

Osteosarcoma (OS) is a devastating primary bone cancer in dogs and humans. Histotripsy is a non-invasive ablation technique that mechanically destroys targeted tumor with focused ultrasound and has shown promise in treating small portions of canine OS followed by limb amputation. This study represents the first use of histotripsy to treat large volumes of canine OS, using fractionated treatments, without subsequent surgical resection. Our objective was to evaluate the safety and feasibility of treating large volumes of OS, following clinical outcomes after histotripsy. 9 dogs with suspected OS were treated with 1–5 histotripsy ablations, depending on tumor size, fractionated over 3 to 17 days. Pain, quality of life, and gait were followed over time, and MRI was used for pre-treatment planning and post-treatment monitoring. All dogs tolerated fractionated histotripsy treatments well, and the ablation zone was visible as a non-enhancing area on MRI matching the targeted location. There was a significant reduction in tumor contrast enhancement after completion of all histotripsy treatments and at the end of follow-up. Across all dogs with follow-up, there was a significant increase in pressure on the tumor-bearing limb during walking at the end of follow-up as compared to pre-treatment, indicating decreased pain. 4/6 dogs with follow-up showed a clinically significant decrease in pain. This study shows the promise of histotripsy as a non-invasive limb-sparing treatment for OS and advances the use of fractionated histotripsy treatments to ablate large and complicated tumors.

**Supplementary Information:**

The online version contains supplementary material available at 10.1038/s41598-026-42319-z.

## Introduction

Osteosarcoma (OS) is the most common primary bone tumor in humans and in canines^1,2^. OS is a devastating diagnosis for both species, with 30% of humans and 90% of canines succumbing to metastatic disease within 5 and 2 years, respectively^3^. The incidence of OS in dogs is estimated to be 10–50 times higher than in humans^4,5^, and combined with the canine’s shortened lifespan, accelerated disease progression, and intact immune system, canine clinical trials for OS are a valuable tool to investigate novel therapies that could benefit dogs and humans. Standard of care in both species involves removal of the primary tumor via limb amputation or salvage surgery, combined with chemotherapy to address metastatic disease^6^. However, limb salvage surgeries have high rates of complications such as deep bone infection, local recurrence, and implant failure, leading to the need for revision surgeries and in some cases, eventual limb amputation^7–9^. Limb salvage and amputation surgeries can impair patient function and mobility^10,11^, and despite these aggressive surgical treatment measures, survival remains poor for both species due to resistant metastatic disease. There is a crucial need for novel limb salvage treatments for OS and other bone tumors that can improve patient quality of life and clinical outcomes.

Histotripsy is a non-invasive focused ultrasound (FUS) ablation modality being investigated for multiple indications, including OS and soft tissue sarcoma (STS)^12–15^. Histotripsy uses short, high-pressure FUS pulses to mechanically destroy targeted tissue with high precision via acoustic cavitation, forming a “bubble cloud” that rapidly expands and collapses to damage surrounding cells^16^. Histotripsy recently received FDA approval for the treatment of liver cancer in humans^17^ and is in clinical trials for renal [NCT05432232], [NCT05820087] and pancreatic cancers [NCT06282809]. Furthermore, thermal high-intensity FUS is FDA approved for palliation of pain from bone metastases^18,19^. However, thermal ablation modalities are limited by the potential for soft tissue burns, damage to surrounding healthy tissues such as nerves and bones, and the heat sink effect which limits treatment efficacy near blood vessels^19–21^. In comparison, histotripsy is tissue-selective and has been shown to spare tissues with higher mechanical strength, such as large vessels^22–24^, bile ducts^22–24^, nerves^25,26^, and healthy bone^26^. Histotripsy has primarily been investigated for soft tissue targets due to the increased resistance of tissues with higher mechanical strength to histotripsy-induced damage^22^, but emerging work indicates that stiffer tissues, including bone tumors, can be ablated with histotripsy. An *ex vivo* feasibility study showed that heterogeneous OS tumors can be ablated with high doses of histotripsy, while normal bone and nerve are spared at the same treatment parameters^26^. This makes histotripsy a natural investigative pathway for the non-invasive treatment of bone tumors.

Canine clinical trials targeting a small portion of OS tumors with histotripsy, followed by surgical resection via limb amputation, have recently shown the *in vivo* safety and efficacy of treating heterogeneous OS with histotripsy. Bone and soft tissue components of OS have been effectively ablated with histotripsy, as assessed by histopathological analysis^13,14^. Although CT imaging is typically used to assess histotripsy ablation in soft tissue targets such as liver and STS^15,17^, CT did not consistently visualize histotripsy ablation in these original canine OS studies^14^. To address this limitation, another recent treat-and-resect *in vivo *study was conducted to investigate the first use of MR imaging to assess histotripsy ablation in canine OS, showing agreement between gross, histopathological, and radiological assessments of ablation^27^. This opens up the potential for long-term treat-and-leave clinical trials of histotripsy for bone tumor ablation.

Until now, our veterinary clinical trials of histotripsy for OS have been limited to treat-and-resect designs targeting only a small portion (typically 4.19 cm^3^) of the entire tumor in a single treatment session. Ablation of large volumes in bone tumors has not previously been accomplished *in vivo *due to device constraints, long treatment durations, and concerns for tumor lysis syndrome^28–30^. The treat-and-resect nature of these prior clinical trials also limits our knowledge of the long-term clinical outcomes after histotripsy in OS patients. The present study is the first reported treat-and-leave clinical trial of histotripsy for canine OS, utilizing higher pulse repetition frequency (PRF) pulsing regimes for faster volumetric ablation of bone tumors. This is also the first published study to use fractionated histotripsy treatments, performed on separate days, in order to achieve maximum ablation of large tumors. Follow-up measurements include veterinarian lameness assessments, gait analysis on a pressure-plate walkway, owner-reported pain and quality of life surveys, and MR imaging to assess treatment safety and efficacy. We hypothesize that fractionated histotripsy treatments can safely ablate large volumes of OS tumors, as evidenced by a lack of contrast enhancement on post-treatment MRI. Furthermore, we hypothesize that histotripsy treatment will not significantly worsen gait, pain, and quality of life in canine OS patients, demonstrating the potential of histotripsy as a non-invasive limb-salvage treatment for OS.

## Methods

### Patient recruitment and veterinary clinical trial design

Pet dogs with suspected appendicular OS (*n* = 9), based on radiographic and/or cytologic evidence, were eligible to enroll in the veterinary clinical trial. To enroll in the trial, owners must have been informed of the clinical trial requirements, including repeat follow-up visits and questionnaires, and they must have declined all standard-of-care treatment options (i.e. limb amputation, radiation, and chemotherapy). Eligibility criteria included no evidence of pulmonary metastases on 3-view thoracic radiographs and no prior tumor-directed treatments. At the time of enrollment, owners were instructed on how to complete 2 questionnaires on behalf of their pet to assess pain and quality of life at home throughout the trial duration. No owner identifying information was present on the questionnaires. Informed and signed owner consent was obtained for all canine patients. All experiments involving animals were approved by the Virginia Tech Institutional Animal Care and Use Committee approval (IACUC protocol #22–091) and were performed according to Animal Research: Reporting of In Vivo Experiments (ARRIVE) regulations and guidelines. Owners were able to withdraw from the study at any time and seek standard of care treatment. Dogs were permitted to stay on their previously prescribed pain medications for the duration of the study, as recommended by the lead veterinarian (J.T.). 1 dog (Patient #8) additionally received a single dose of zoledronate (anti-osteoclast) 14 weeks after the initial histotripsy treatment.

**Fig. 1:**
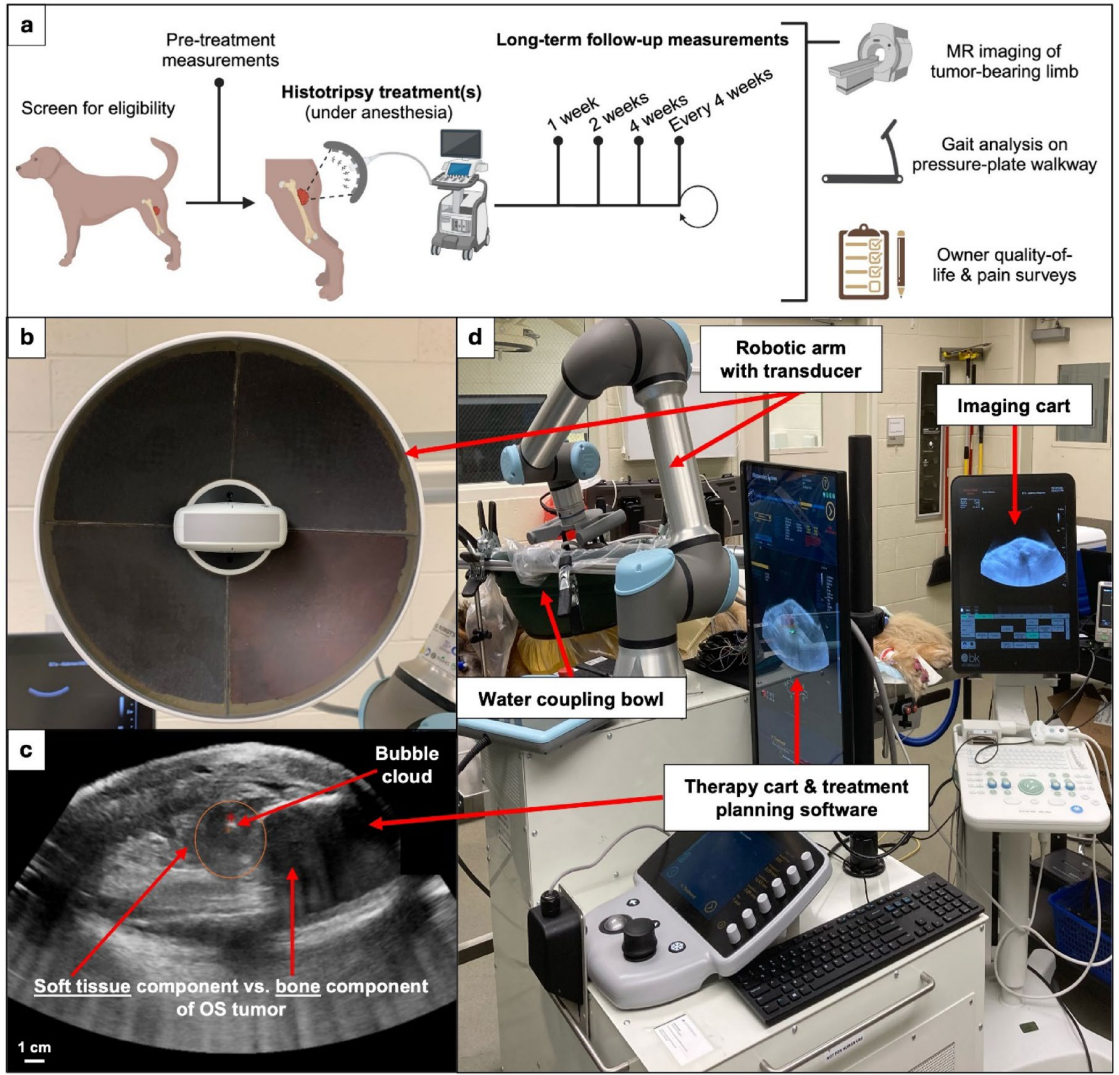
Clinical trial workflow and canine histotripsy set-up for the treatment of appendicular OS. (**a**) Canine clinical trial workflow. (**b**) 700 kHz histotripsy transducer with coaxially aligned 1–5 MHz curvilinear US imaging probe. (**c**) Real-time US imaging during histotripsy ablation, showing the cavitation bubble cloud and the bone versus soft tissue components of a representative OS tumor. (**d**) Overall set-up for histotripsy treatment of canine appendicular OS, where the transducer is mounted on a triple-jointed robotic arm and coupled to the prepared limb with a bowl containing degassed water. Figure 1A was created using Biorender.com.

After enrollment, dogs received a pre-treatment MRI to aid in treatment planning, followed by histotripsy treatment. Multiple treatment volumes, fractionated over days or weeks, were applied to ablate as much tumor volume as possible. If residual or new tumor growth was noted on MRI, the decision for subsequent treatments after the initial planned treatment(s) was made based on the owner’s wishes and clinician assessment. After and throughout the course of histotripsy treatments (in cases with multiple treatments), canine patients underwent follow-up data collection at 1, 2, and 4 weeks after the initial histotripsy treatment, as well as every 4 weeks after that until the patient’s disease had progressed such that the dog was unable to travel or the owner elected for euthanasia. Euthanasia for these canine patients was performed using an approved method by the American Veterinary Medical Association involving an intravenous overdose of barbiturate at a dose of 1 mL per 5 kg of body weight. The overall trial workflow is outlined in Fig. 1A.

### Histotripsy system and treatment.

The histotripsy workflow used in this canine clinical trial is a next-generation version of the system in our previous treat-and-resect clinical trials^13,14,27^ and offers improved robotic targeting and imaging guidance, as shown in Fig. 1D. A high-powered 32-element 700 kHz histotripsy transducer with an aperture of 18 cm, a focal length of 11 cm (IMASONIC SAS, Voray-sur-l’Ognon, France, Fig. 1B), and a central cut-out for an ultrasonic imaging probe was used to send therapy pulses and provide real-time US imaging guidance during treatment (Fig. 1C). The transducer and coaxially-aligned 1–5 MHz curvilinear ultrasonic imaging probe (5C1e Curved Array Transducer, BK Medical, Herlev, Denmark) were integrated onto a prototype clinical system (HistoSonics, Ann Arbor, MI, USA). The therapy/imaging complex was mounted on a triple-jointed robotic arm (UR5e, Universal Robots, Odense, Denmark) that could be controlled through custom treatment planning software (HistoSonics, Ann Arbor, MI, USA). The histotripsy transducer was calibrated in free-field with a fiber optic hydrophone (HFO-50, ONDA, Sunnyvale, CA, USA).

All dogs were placed under general anesthesia maintained with inhaled isoflurane for the entirety of the histotripsy treatment, and vital signs (temperature, heart rate, blood pressure, respiration, oxygen saturation, and electrocardiogram waveforms) were continuously monitored by a licensed veterinary technician with veterinarian oversight. Once the patient was induced, the hair overlying the planned treatment location was removed with clipping and shaving followed by application of a depilatory cream (Nair™, Church & Dwight Co. Inc., Ewing, NJ, USA) for 5–15 min, depending on the thickness and texture of each dog’s coat. After hair removal, the planned treatment site was coupled for acoustic propagation by placing a container of degassed water attached to the patient using an Ioban™ surgical drape cut to custom-fit each patient’s targeted treatment zone, with an opening in the drape such that the water was directly in contact with the patient’s skin. Freehand US imaging was performed to guide pre-treatment targeting, and the transducer was positioned in the coupling bath such that the focus of the transducer was aligned with the planned treatment location. Patient-specific ablation plans were developed based on pre-treatment MR images. The region of interest for treatment was identified on the limb by measuring from a grossly palpable landmark (e.g. carpal pad, tibial crest) to the center of the planned ablation volume.

To achieve ablation of large tumor volumes, higher PRF histotripsy pulsing regimes were used in this study as compared to our prior studies. The treatment volume contained a 3D grid of equidistant treatment points (spaced 3.36 mm in the axial direction and 1.08 mm in the lateral and elevational directions based on the dimensions of the focused beam). For the first 3 dogs treated, histotripsy was delivered at a PRF of 1500 Hz and a dosage of 750 pulses per point (ppp). After suspected thermal trauma in 2 dogs at those parameters, pulsing regimes were scaled back to a PRF of 1000 Hz and a dosage of 500 ppp for the following 6 dogs. 1 patient in this second cohort (Patient #6) had a final treatment at 750 ppp after potential new tumor growth on MRI. For these 6 dogs, a circulating cooling pad (Polar PupⓇ Crate and Bed Cooler, Polar Products, Stow, OH, USA) was placed on the post-focal surface of the skin, and temperature was monitored using Type T thermocouples positioned on the skin surface (TC-08, Omega Engineering Inc., Norwalk, CT, USA). The cooling pad included a reservoir filled with iced water and reached temperatures of ≤ 10 °C on the skin surface; to avoid any potential hypothermic effects, the cooling pad followed a 10 min on – 10 min off cycle repeated throughout the duration of histotripsy treatment.

For each patient, the mean treatment pressure was determined via 7-point test pulsing (at the center and each boundary of the spherical or elliptical ablation volume). The treatment pressure at each point was chosen by increasing pressure incrementally until cavitation was identified on US B-mode imaging. In cases where the bubble cloud was not visible beyond intact cortical bone, the treatment pressure was increased as much as possible before pre-focal cavitation was observed on the skin surface.

### Adverse event reporting

Adverse events related to histotripsy treatment were recorded using the Veterinary Cooperative Oncology Group Common Terminology Criteria for Adverse Events (VCOG-CTCAE v2)^31^.

### MRI acquisition and imaging assessments

Before histotripsy and over time after treatment, MR images were acquired with either a 1.5T Philips Intera scanner (first 3 dogs) or a 3 T Siemens MAGNETOM Vida scanner (last 6 dogs). For the 1.5T scanner, sequences followed our past work characterizing MRI for histotripsy ablation in OS^27^ and included the following: dual-echo T2- and proton density-weighted (DE T2w and PD, respectively), short T1 inversion recovery (STIR), variable density incoherent spatiotemporal acquisition (VISTA), and pre- and post-contrast 3D T1-weighted turbo field echo (T1w TFE) and T1-weighted fat-saturated (T1w FS). For the 3 T scanner, sequences included DE T2w and PD, STIR, SPACE, and pre- and post-contrast T1w FS DIXON and T1w VIBE images. DIXON images were acquired as in-phase, out-of-phase, fat-only (DIXON_F), and water-only (DIXON_W). T1w images were acquired prior to and following the intravenous administration of a gadolinium-based contrast agent (ProHance^®^ from BRACCO Diagnostics, Monroe, NJ, USA). All images were acquired in the sagittal plane.

Qualitatively, MR images were assessed by a board-certified veterinary radiologist (G.B.D., 35 years of experience), a human diagnostic and interventional radiologist with extensive experience in assessing ablation on imaging (T.J.Z., 18 years of experience), and 2 human musculoskeletal radiologists (S.B.S. and G.B.M., 14 and 3 years of experience, respectively). Radiologists focused on assessments of signal intensity and enhancement patterns within and around the tumor and the treatment zones, including looking for potential off-target effects.

Quantitatively, canine OS tumors were segmented from contrast-enhanced T1w FS images (DIXON_W for the 3 T MRI scanner) using the open-source software 3D Slicer (www.slicer.org), following previous work in MR analysis of canine OS^27^. Segmentations were performed by the primary author (E.R.V., 3 years of experience) and a veterinary student (J.S.K., 1 year of experience), and the reported volumes and signal intensities are averaged between the 2 observers. The whole tumor volume was segmented using the grow from seeds method. Quantitative MRI data is reported only for the 4 dogs with follow-up images on the 3 T scanner (> 50% ablation based on pre-treatment tumor volume on MRI).

Tumor enhancement was quantified on DIXON_W images by first subtracting the pre-contrast tumor signal intensity from the post-contrast tumor signal intensity. To normalize the signal intensity of the tumor for each imaging session, a spherical volume (> 1000 voxels and > 1 cm^3^) was segmented in the healthy muscle adjacent to the tumor. The spherical volume was chosen to be as close to the tumor and treatment zones as possible (< 10 cm) while selecting a homogenous region of muscle tissue. The tumor signal intensity was then divided by the signal intensity of the adjacent healthy muscle (post-contrast muscle signal – pre-contrast muscle signal) to yield normalized tumor enhancement.

### Gait analysis and clinical lameness assessments

Before histotripsy and over time after treatment, canine OS patients were walked across a low-profile pressure-plate walkway (Animal Strideway™ System, Tekscan Inc., Norwood, MA, USA). The walkway was calibrated at a range of weights (9–59 kg), and each dog’s gait was calibrated based on the dog’s weight. Dogs were walked at an optimal velocity of 80–120 cm/s, and animal handlers were kept consistent between walking sessions when possible. Each dog had 4–7 walkway trials per timepoint, depending on how tolerant each dog was of the walkway. Gait analysis parameters were averaged across all trials per timepoint and per dog. Peak pressure, stance time, body weight distribution, and vertical impulse distribution were extracted for the tumor-bearing limb and compared between the start of the study and the end of the follow-up period for each dog and averaged across all dogs. Additionally, lameness was scored 0–5 by a board-certified veterinary surgical oncologist (J.T.) at every patient visit. The lameness scale is as follows: 0 = no detectable lameness at any gait; 1 = barely perceptible lameness; 2 = mild or inconsistently apparent, weight-bearing lameness; 3 = moderate, obviously apparent, weight-bearing lameness; 4 = severe, predominantly weight-bearing lameness; 5 = severe, predominantly non-weight-bearing lameness.

### Pain and quality of life assessments

Before histotripsy and over time after treatment, owners were instructed to complete 2 questionnaires on behalf on their pet to assess their dog’s pain and quality of life at home. The Canine Owner-Reported Quality of Life (CORQ)^32^ survey is validated in dogs with cancer undergoing treatment, and the Canine Brief Pain Inventory (CBPI)^33^ is adapted from the Brief Pain Inventory in human medicine and has been validated for dogs with bone cancer. The CORQ (0–7 scale, with 7 signifying maximum quality of life or pain) has 5 items to assess vitality, 6 items for companionship, 4 items for mobility, and 2 items for overall pain (doi:10.2460/javma.252.9.1073, Supplementary Appendix S2). The CBPI (0–10 scale, with 10 signifying maximum pain) has 5 items to assess pain severity and 6 items to assess pain interference (vet.upenn.edu/ryan-hospital/clinical-trials/pennchart/). Scores for each parameter were compared at all patient visits and are reported at the start of the study and the end of the follow-up period for each dog and averaged across all dogs.

### Statistical analysis

All statistical analyses were performed using GraphPad Prism (version 10.1.1). Statistical significance was defined as *P* < 0.05. The residuals of all datasets being used for t-tests were tested for normality using the Shapiro-Wilk, Anderson-Darling, Kolmogorov-Smirnov, and D’Agostino-Pearson tests; the residuals were considered to follow a normal distribution if *P* > 0.05 for at least 2/4 normality tests. If the residuals did not follow a normal distribution, the non-parametric statistical test equivalent was used. All reported descriptive statistics represent mean ± standard deviation unless otherwise stated.

Pearson’s correlations were used to test the relationship between individual observer segmentations of OS tumors on MRI (E.R.V. and J.S.K.). Inter-observer variability was determined to be acceptable if there was a significant correlation between observers.

For gait analysis parameters, to assess the overall patient response, paired t-tests or the non-parametric equivalent were used to compare each parameter averaged across all dogs between the start of the study and the end of the follow-up period. To assess individual patient responses, individual walkway trial replicates were compared between timepoints for each dog using unpaired t-tests or the non-parametric equivalent. For the CBPI, a 1-score reduction in pain severity and a 2-score reduction in pain interference were considered clinically significant, following the guidelines for using this assessment tool^33,34^.

## Results

### Patient population and histotripsy treatments

9 dogs were treated with histotripsy in this study, with 6 dogs receiving follow-up. Across those 9 dogs, 24 histotripsy treatments were delivered. Patient characteristics are outlined in Table 1. At the time of enrollment, the mean age and weight of canine patients were 6.2 ± 2.9 years and 47.8 ± 24.1 kg, respectively. Mean tumor volumes based on baseline pre-treatment MR images were 112 ± 147 cm^3^. 5/9 patients were definitively diagnosed with OS based on histopathological analysis of the limb after owner-elected amputation or euthanasia. For 2/9 patients where the tumor-bearing limb was unable to be acquired, the bone lesion was suspected to be OS based on radiographic appearance. Patient #1 was initially diagnosed as OS based on radiographic appearance and a fine needle aspirate, but histopathology of the tumor-bearing limb after euthanasia revealed a round cell neoplasm with bone lysis of the affected scapula.

**Table 1:** Patient demographics and bone tumor characteristics for all patients enrolled in the present study.

Patient	Breed	Age (years)	Weight (kg)	Tumor location	Tumor type	Baseline tumor volume (cm^3^)	Follow-up period (weeks)
1	Labrador Retriever	1	27	Scapula	Round cell neoplasm	129	4
2	Great Dane	5	74	Distal radius	Suspected osteosarcoma*	52	2
3	Mixed Breed	11	22	Distal radius	Osteosarcoma	5	N/A
4	Leonberger	4	62	Distal radius	Osteosarcoma	101	6
5	Golden Retriever	6	38	Scapula	Osteosarcoma	489	N/A
6	Mixed Breed	8	32	Distal tibia	Osteosarcoma	30	8
7	Leonberger	6	94	Proximal tibia	Suspected osteosarcoma*	87	6
8	Rhodesian Ridgeback	6	46	Distal tibia	Osteosarcoma	28	22
9	American Bully	9	35	Distal radius	Osteosarcoma	42	N/A

*Suspected osteosarcoma indicates that the tumor was presumed to be OS based on radiologic appearance but was not able to be confirmed post-mortem. For Patient #2, a urine fungal antigen test ruled out fungal osteomyelitis. For Patient #7, a fine needle aspirate indicated sarcoma, suspected to be OS due to location of tumor and cell morphology. Patient #1 was initially diagnosed as OS based on radiographic appearance and a fine needle aspirate, but histopathology of the tumor-bearing limb after euthanasia revealed a round cell neoplasm with bone lysis of the affected scapula. N/A indicates lost to follow-up due to withdrawal from study.

In general, patients recovered well after treatment and were able to be discharged to their owners within 1–3 hours post-histotripsy. The mean follow-up period was 8.1 ± 7.1 weeks, ranging from 2 to 22 weeks post-enrollment. 3 dogs were withdrawn from the study and therefore had no follow-up data: Patients #3 and #9 elected for limb amputation, and Patient #5 was euthanized due to large tumor volume (489 cm^3^, nearly 300% larger than the next largest tumor treated in this study).

Table 2 outlines the histotripsy treatment characteristics for all dogs in this study. Full details for each individual treatment are given in Supplementary Table S1. Canine patients were treated with 1–5 histotripsy treatment volumes (2.7 ± 1.8 treatments), with the aim of multiple treatments being to achieve a larger overall volume of tumor ablation. This typically occurred in tumors that were relatively large (> 30 cm^3^) at the time of enrollment. Treatments were fractionated across days or weeks, with the shortest time in between primary treatments being 3 days and the longest time being 17 days. For re-treatments of previously treated regions, the shortest time from the final primary treatment to the re-treatment was 16 days, and the longest time was 34 days. The smallest treatment volume (4.7 cm^3^) had a therapy time of 40 min, while the largest treatment volume (29.2 cm^3^) had a therapy time of 240 min. On average, individual treatment volumes took 111.4 ± 60 min to complete, and the total treatment duration was 290.0 ± 227.4 min. Volumes of 14.1 ± 7.5 cm^3^ were ablated per treatment, with a total treatment volume of 35.6 ± 27.5 cm^3^.

**Table 2:** Histotripsy treatment parameters and characteristics for all canine patients treated in the present study.

Patient	Number of treatment volumes	Longest treatment dimension (cm)	Average peak negative pressure (MPa)	Average therapy duration per treatment (min)	Total therapy duration (min)	Average ablation volume per treatment (cm^3^)	Total ablation volume (cm^3^)	Ablation coverage of tumor (%)
1	1	3.0	33.2	60	60	9.8	9.8	4.9
2	2	3.5	47.9	100	200	12.0	23.9	25.0
3	1	4.0	39.5	40	4012	4.7	4.7	98.1
4	4	6.0	43.9	135	540	15.5	62.0	60.6
5	1	3.5	34.1	240	240	29.2	29.2	6.0
6	5	5.0	42.0	75	350	7.3	36.6	100.6*
7	5	5.5	46.7	143	715	18.5	92.7	74.4
8	4	5.0	40.4	90	360	10.5	41.9	153.3*
9	1	5.0	45.7	120	120	19.6	19.6	46.1
Mean$\pm$ standard deviation	2.7$\pm$1.8	4.5$\pm$1.1	41.5$\pm$5.3	111.4$\pm$60.0	290.0$\pm$227.4	14.1$\pm$7.5	35.6$\pm$27.5	63.2$\pm$49.1

*Patients #6 and #8 are reported to have over 100% ablation coverage of the tumor volume due to overlapping treatments in the same area.

**Fig. 2:**
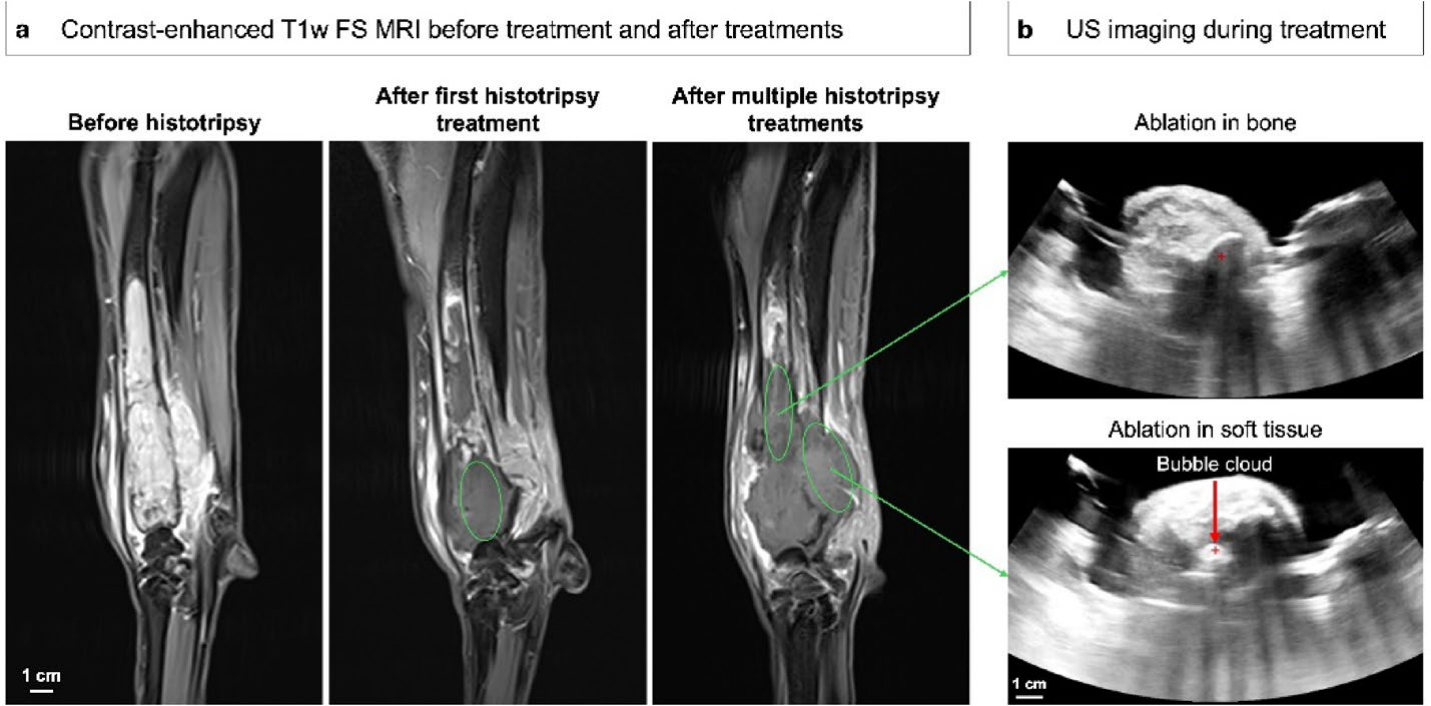
MR imaging before and after multiple histotripsy treatments, along with US imaging during treatments, in a 4-year-old female intact Leonberger (Patient #4). (**a**) Contrast-enhanced T1w FS MRI before histotripsy treatment, after the first treatment, and after two additional treatments. The green circles indicate the planned treatment regions. (**b**) Real-time US imaging corresponding to ablation targeted in bone (top) versus soft tissue (bottom) regions of OS. The cavitation bubble cloud is only visible in soft tissue.

Patients were treated at a mean peak negative pressure of 41.5 ± 5.3 MPa, with lower pressures often being used in soft tissue portions of tumors and higher pressures often used in areas with more intact cortical bone. Patient-specific treatment pressures were determined by incrementally increasing pressure at the focus in 7 points throughout the treatment volume until cavitation was achieved or until continuous pre-focal cavitation was observed on the skin. Figure 2 shows representative US images from 1 patient (Patient #4) with treatments targeted in the soft tissue portion vs. in the bone-encased medullary cavity of the OS tumor. The histotripsy bubble cloud was generally visible on real-time US imaging when targeting in softer portions of OS tumors or in areas with little intact cortical bone (Fig. 2 C), but not when treating deep in the bone or beyond intact cortical bone (Fig. 2B). For all patients regardless of bubble cloud visibility during treatment, post-treatment MRI was used to confirm ablation in the targeted region for each planned treatment.

Ablation coverage of the tumor was determined for each patient by dividing the targeted ablation volume by the whole tumor volume on the most recently acquired MRI (Table 2). The average percent ablation was 63.2 ± 49.1%, with 4/6 dogs with follow-up receiving greater than 50% ablation coverage of their tumor volume (Patients #4, #6, #7, & #8). 2 of those dogs (Patients #6 & #8) are reported to have > 100% ablation coverage of the tumor volume due to multiple treatments overlapping in the same area. Residual untreated tumor was localized to the tumor periphery.

### MR imaging over time

**Fig. 3:**
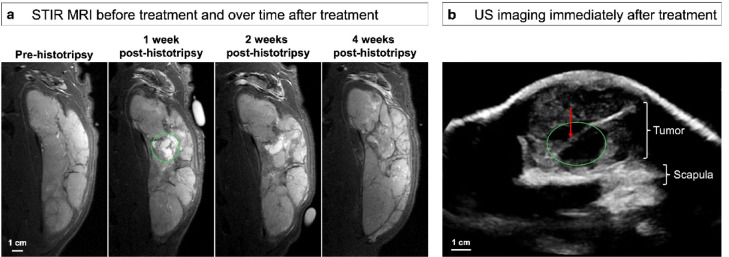
MR imaging before and over time after histotripsy treatment, along with US imaging during treatment, in a 1-year-old female intact Labrador Retriever (Patient #1). (**a**) STIR MRI before the histotripsy treatment and 1, 2, and 4 weeks post-histotripsy. The treatment zone is indicated in a green circle as a hyperintense (fluid-filled) region. (**b**) Real-time US imaging after histotripsy treatment showing the hypoechoic treatment zone (green circle) with a suspected intact fascial plane running through the treated area (red arrow).

**Fig. 4:**
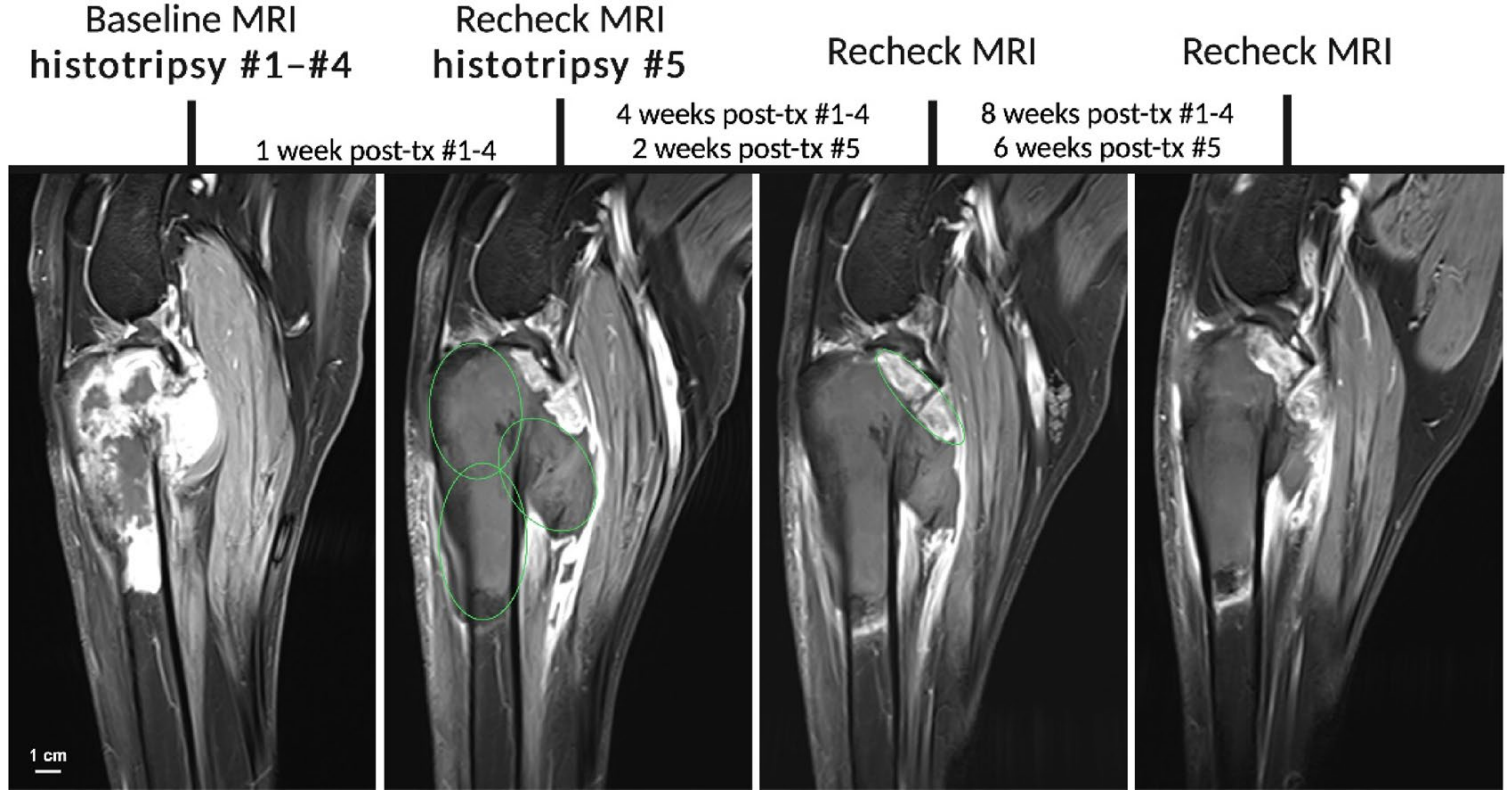
Contrast-enhanced T1w FS MR imaging before and over time after histotripsy treatments in a 6-year-old male neutered Leonberger (Patient #7). The planned treatment regions are indicated by green circles. The final treatment volume does not show a lack of contrast enhancement after treatment likely due to blockage by the head of the fibula, inhibiting an effective ablation. Tx = treatment.

MR images were acquired before treatment and over time during and after histotripsy treatments. For all canine patients, the treatment zone was visible as a hypo-intense, non-enhancing volume matching the planned treatment location on contrast-enhanced MRI. The non-enhancing area persisted up to 4–8 weeks post-histotripsy. For treatments targeted in softer portions of bone tumors, the ablation could be visualized as an area of increased signal intensity on fluid-sensitive sequences such as T2w and STIR images. STIR images over time are shown for Patient #1, who had a primarily soft tissue tumor, in Fig. 3. This patient received a single treatment that was well-localized on STIR MRI and resorbed by 4 weeks post-histotripsy (Fig. 3A). On real-time US imaging during treatment, the ablation was visible as a hypo-echoic zone with an intact fascial plane running through the treated volume (Fig. 3B). Contrast-enhanced T1w FS images are shown over time in Fig. 4 for Patient #7, who showed only a 5.8 cm^3^ increase in size of the residual untreated tumor over the course of 8 weeks. For this patient, the remaining residual tumor was targeted in a 5th treatment volume 1 week after the original treatments, but an effective ablation was not visualized on post-treatment MRI.

Qualitatively, out of the 6 dogs with follow-up imaging data, 2/6 dogs did not show evidence of tumor growth (new contrast enhancement) on the final image as compared to the pre-treatment image. These final follow-up images were taken at 2 weeks post-treatment (Patient #2) and 8 weeks post-treatment (Patient #7). The remaining 4 dogs eventually showed changes in contrast enhancement at the periphery of the tumor that are suggestive of tumor growth over time. Additionally, 2/6 dogs showed a potential extension of ablative effect after histotripsy treatment, showing a loss of contrast enhancement in an area outside of the targeted ablation zone. A representative loss of contrast enhancement in the medullary cavity after a nearby histotripsy ablation is shown in Fig. 2A. No off-target damage or ablation was noted on MR imaging.

**Fig. 5:**
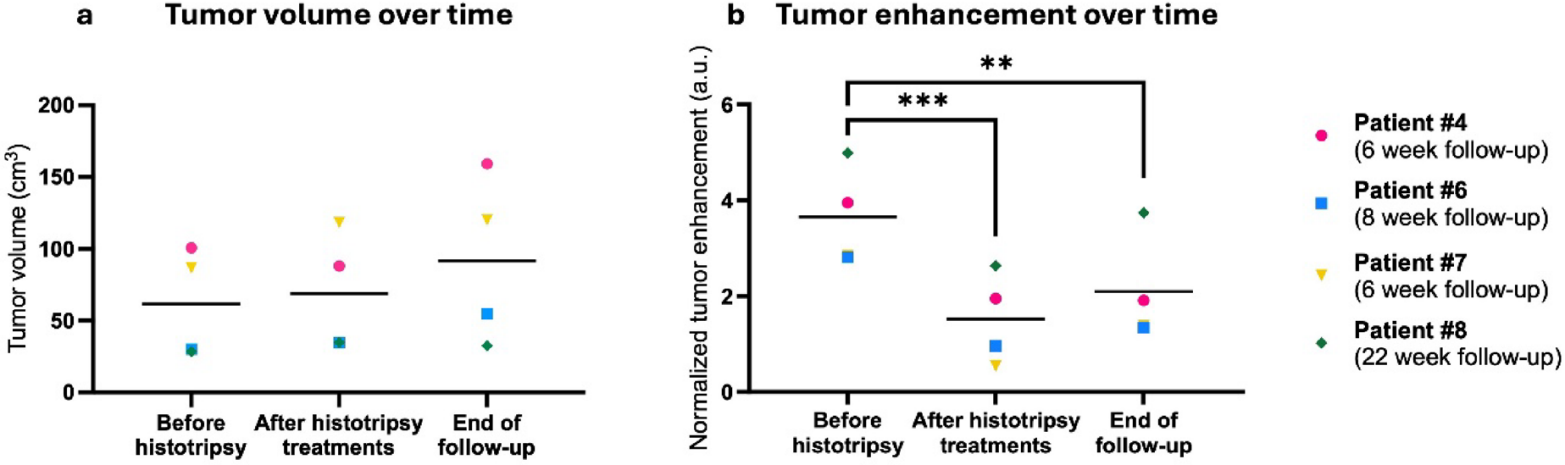
Quantitative MRI data over time in 4 canine OS patients that received histotripsy treatment to > 50% of their tumor volume with long-term follow-up. (**a**) Tumor volume over time. (**b**) Tumor enhancement over time (post-contrast tumor signal intensity – pre-contrast tumor signal intensity) normalized to healthy muscle (post-contrast muscle signal intensity – pre-contrast muscle signal intensity).

Quantitatively, tumor volume and enhancement are shown over time in Fig. 5 for the 4 dogs with follow-up that received greater than 50% ablation coverage based on pre-treatment MRI tumor volumes. Whole tumor volumes for all dogs except 1 (Patient #4) increased slightly after the completion of all histotripsy treatments (mean of 11.9% increase in tumor volume across all dogs, Fig. 5A). Additionally, all dogs had larger tumor volumes at the end of follow-up as compared to before treatment, ranging from an increase in tumor volume of 13.7% (Patient #8) to 80.4% (Patient #4). From the completion of all treatments to the end of follow-up, 1 dog showed a slight 1.6% increase in tumor volume (Patient #7), while 2 dogs showed a larger increase in tumor volume (mean of 69.3% increase). Another dog showed a 6.6% decrease in tumor volume over the 17 weeks after the completion of all treatments (Patient #8, Fig. 5A). Considering whole tumor enhancement patterns, dogs showed a significant decrease in enhancement after all treatments (58.2% decrease, *P* = 0.0009) and at the end of follow-up (42.6% decrease, *P* = 0.0055) as compared to pre-treatment (Fig. 5B). All tumor segmentations were performed and averaged between 2 observers, and Supplementary Fig.S1 shows the correlations between observers for each patient’s whole tumor segmentation. All correlations were significant (*P* < 0.05), indicating good agreement between observers.

### Clinical outcomes after histotripsy treatments

Lameness, gait, pain, and quality of life were assessed before treatment(s) and over time after treatment(s). Supplementary Fig. S2 includes videos showing 1 dog walking with a marked decrease in lameness 1 month after histotripsy (Patient #6, lameness score of 5 pre-treatment and 0 post-treatment), and Supplementary Fig. S3 includes videos showing 1 dog walking with a mild decrease in lameness 1 month after histotripsy (Patient #7, lameness score of 2 pre-treatment and 1 post-treatment).

**Fig. 6:**
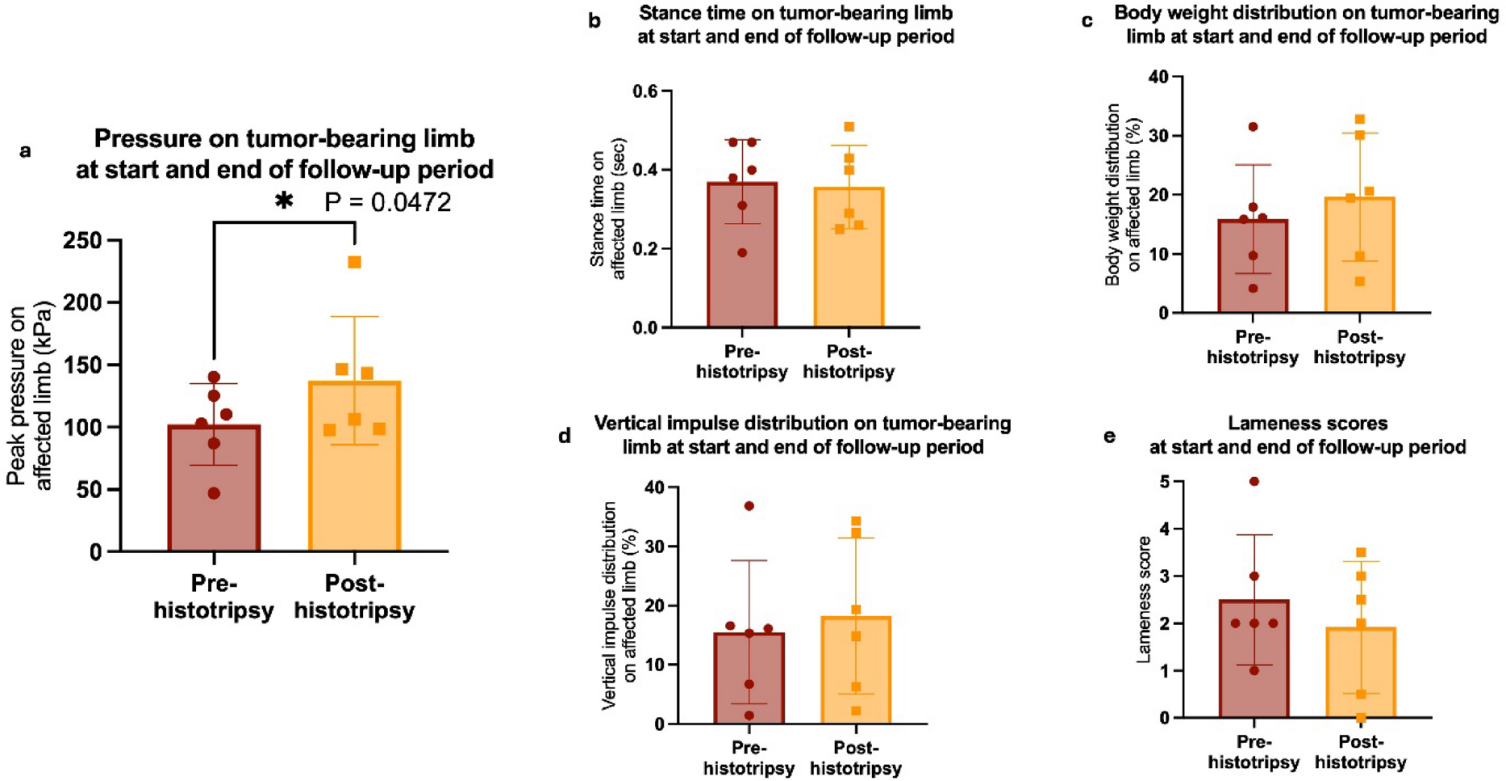
Gait analysis and lameness parameters across all 6 dogs with follow-up data in the present study, showing mean $\pm$ standard deviation at the start and end of each dog’s follow-up periods. * indicates statistical significance (*P* < 0.05) as determined by paired t tests. (**a**) Peak pressure on tumor-bearing limb. (**b**) Stance time on tumor-bearing limb. (**c**) Body weight distribution percentage on tumor-bearing limb. (**d**) Vertical impulse distribution percentage on tumor-bearing limb. (**e**) Clinical lameness scores.

Gait outcomes averaged across all 6 dogs with follow-up data are shown in Fig. 6, with outcomes per individual patient and overall statistics shown in Supplementary Table S2. On pressure-plate walkway analysis, comparing from the start of the study (before treatment) to the end of each dog’s follow-up period, canine patients were putting significantly more pressure on their tumor-bearing limb after treatment (*P* = 0.0472, Fig. 6). For all other analyzed gait parameters, including stance time, body weight distribution, and vertical impulse distribution on the tumor-bearing limb, there was no significant difference between pre- and post-histotripsy assessments (*P* > 0.05 for all parameters). Body weight distribution and vertical impulse distribution trended in the direction of improvement (more weight on the tumor-bearing limb after treatment), while stance time remained the same (mean stance time of 0.4 s before and after treatment, Supplementary Table S2). Similarly, there was no significant difference in lameness score before and after treatment (*P* > 0.05, Supplementary Table S2), although there was a trend towards a lower lameness score after treatment. Considering individual canine patients, Patient #2 had a significant improvement from the start to the end of the follow-up period (2 weeks) in peak pressure (*P* = 0.0287), body weight distribution (*P* = 0.0224), and vertical impulse distribution (*P* = 0.0286) on the tumor-bearing limb (Supplementary Table S2). All other patients did not show significant differences between pre- and post-histotripsy assessments (*P* > 0.05 for all parameters).

**Fig. 7:**
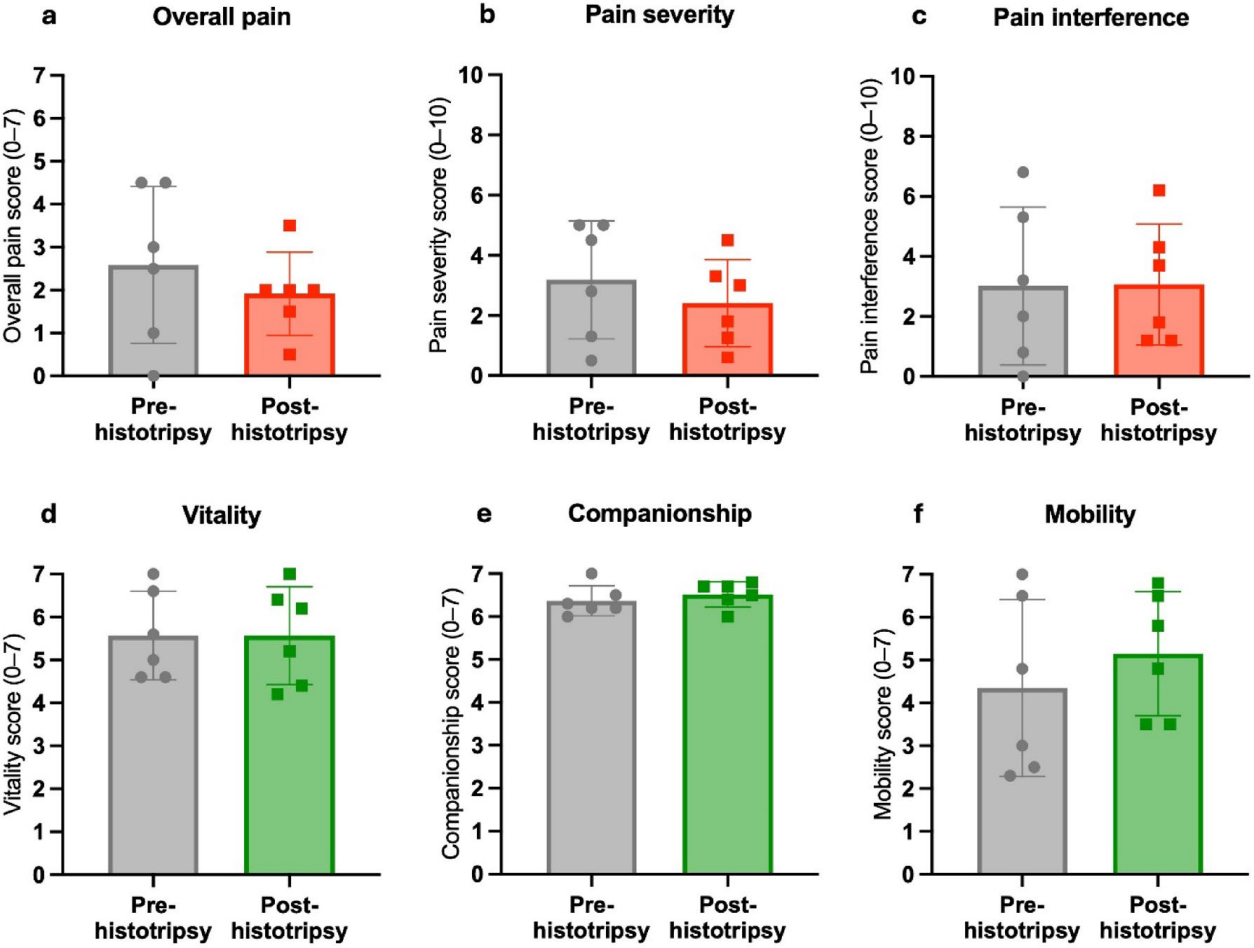
Pain and quality of life scores across all 6 dogs with follow-up data in the present study, showing mean $\pm$ standard deviation at the start and end of each dog’s follow-up periods. All changes were non-significant (*P* > 0.05) based on paired t-tests. (**a**) Overall pain (CORQ, 0–7). (**b**) Pain severity (CBPI, 0–10). (**c**) Pain interference (CBPI, 0–10). (**d**) Vitality (CORQ, 0–7). (**e**) Companionship (CORQ, 0–7). (**f**) Mobility (CORQ, 0–7).

**Fig. 8:**
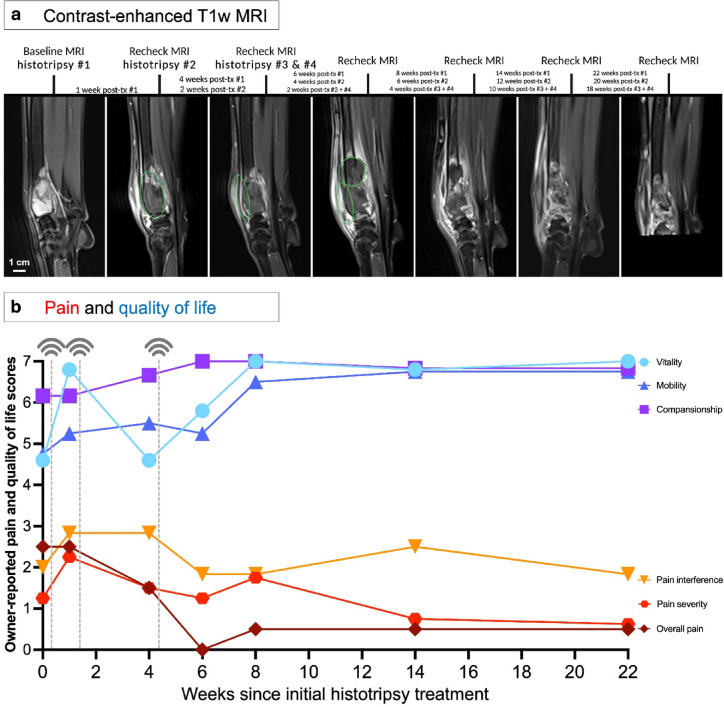
Case study of a 6-year-old male neutered Rhodesian Ridgeback (Patient #8) treated in the present study, with the longest follow-up time (22 weeks since the initial treatment). (**a**) Contrast-enhanced T1w FS MRI over time, with planned treatment regions indicated by green circles. (**b**) Pain and quality of life scores over time, with the dashed vertical lines indicating time of histotripsy treatment.

Considering pain and quality of life assessments via the CBPI and CORQ owner questionnaires, there were no significant differences across all dogs from the start to the end of the follow-up period (*P* > 0.05 for all parameters, Supplementary Table S3). Pain and quality of life outcomes averaged across all 6 dogs with follow-up data are shown in Fig. 7. All parameters trended in the direction of improvement (lower pain and higher quality of life after treatment) except for vitality on the CORQ and pain interference on the CBPI, which remained at the same mean score before and after treatment. Considering individual canine patients, 3/6 dogs showed a clinically significant improvement on the CBPI in pain severity (Supplementary Table S3). A different dog (Patient #2) also showed a clinically significant improvement in pain interference (CBPI). 1 patient (Patient #8, over 100% ablation based on MRI) had the longest follow-up period at 22 weeks post-treatment. Contrast-enhanced T1w FS MRI as well as pain and quality of life scores over time are shown for this patient in Fig. 8, trending towards higher quality of life and lower pain sustained after treatments.

In 24 histotripsy treatments across 9 patients, 4 adverse events related to treatment were recorded (16.7%). All adverse events consisted of varying degrees of injury to the post-focal skin and/or soft tissues. Grade 3 skin ulcerations were recorded in 2 patients, as well as a grade 1 skin ulceration and a grade 1 erythema in 2 other patients^31^. The grade 1 skin ulceration is shown in Supplementary Fig. S4. Patient #3 (grade 3 skin ulceration) withdrew from the study as the owner elected for limb amputation, and the remaining adverse event injuries healed well over time. 2 additional adverse events occurred while the patients were in the care of their owners and were deemed unlikely to be due to the histotripsy device or treatment. 5 days after the second histotripsy treatment (19 days since the initial treatment), Patient #2 had an acute activity-induced cranial sub-luxation of the tumor-bearing limb after the owner reported that the dog stumbled and tripped during urination. 30 days after the second and third histotripsy treatment volumes (41 days since the initial treatment), Patient #6 sustained an acute activity-induced presumptive soft tissue injury after jumping into a leaf pile.

At the time of manuscript preparation, 2/9 dogs had received limb amputation (Patients #3 and #9). Neither patient elected for adjuvant chemotherapy or serial chest radiographs after amputation, so the presence of potential lung metastases is unknown. Patient #9 developed a liver mass and was subsequently euthanized around 24 weeks post-treatment due to this new diagnosis. Patient #3 was euthanized 1 year post-treatment due to increased respiratory effort that was presumed by the primary veterinarian to be associated with potential metastatic disease. The 6 dogs with follow-up whose owners elected against limb amputation were all eventually euthanized due to primary or metastatic disease progression. Survival for these dogs ranged from 38 days (Patient #1, 4.9% ablation of 129 cm^3^ tumor) to 173 days (Patient #8, over 100% ablation of a 28 cm^3^ tumor). Out of these 6 patients, 2 owners (Patients #6 & #8) elected to perform chest radiographs over time to monitor for metastatic disease. The time to metastatic progression for these patients was 14 weeks (#6) and 22 weeks (#8).

## Discussion

To our knowledge, this is the first study to perform the following: fractionated histotripsy treatments to accomplish ablation of large tumor volumes over time; histotripsy ablation of bone tumors without subsequent standard-of-care surgical resection; and histotripsy ablation of large bone tumor volumes (up to 30 cm^3^ in a single treatment). These treatments occurred in a canine comparative oncology model of OS with the goal of advancing OS treatment for both veterinary and human patients.

Overall, fractionated histotripsy treatments were successful in ablating canine bone tumors, as indicated on post-treatment contrast-enhanced T1w FS MRI. The ablation zone for each treatment was visualized as a non-enhancing region suggestive of ischemia and non-perfusion. In soft tissue portions of OS tumors and in a patient with round cell neoplasm (Patient #1), ablation was hyperintense on fluid-sensitive T2w and STIR images, indicating hemorrhage and edema. Only 2 out of 24 ablations were not immediately obvious on post-treatment MRI: 1 proximal tibia ablation in Patient #7 that was likely blocked by the head of the fibula, and 1 very superficial ablation in Patient #8 that was likely limited by shielding from pre-focal cavitation. Otherwise, the targeted ablation matched the size and location of the non-enhancing area visualized on MRI.

Histotripsy ablation on MRI was characterized by a substantial loss of contrast enhancement (58.2% decrease) and a slight enlargement in whole tumor volume (11.9% increase) as compared to pre-treatment. Whole tumor volume increase immediately after histotripsy is likely attributed to swelling and inflammation from the ablation. Whole tumor enhancement showed a significant decrease after completion of all histotripsy treatments and remained significantly lower than pre-treatment at the end of the follow-up period for each dog, suggesting that the tumor was not as vascularized or viable as before the ablation(s) despite having a larger tumor volume. This is similar to what is seen as a response to chemotherapy and radiation therapy in human OS, where literature reports larger post-treatment tumor volumes but decreased enhancement that correlates to tumor necrosis on histopathology^35^.

4/6 dogs with imaging follow-up showed eventual contrast enhancement suspected to be tumor growth at the periphery of the tumor. While it is difficult to discern between untreated tumor growth or ablated tumor regrowth, changes in contrast enhancement were localized mainly at the periphery of the ablation zone and/or tumor and suggestive of untreated peripheral tumor growth and infiltration at the tumor margins rather than regrowth of ablated tumor tissue. This is promising for the clinical translation of histotripsy for OS, suggesting that ablating with a healthy tissue margin and/or incorporating combination therapies may help to eliminate continued growth of residual untreated tumor. Excitingly, 2/6 dogs showed potential extension of ablative effect (loss of enhancement) to untreated regions with the primary targeted tumor, suggestive of a local immunogenic and/or anti-tumor immune response to histotripsy ablation. The first-in-human use of histotripsy for liver cancer saw 2/8 patients show a decrease in cancer biomarkers over time after histotripsy, and 1 of those patients saw a decrease in size of multiple untreated lesions in the liver, also suggestive of a local immune response^17^. These findings highlight the promise of histotripsy as a potential immunomodulator and promoter of an anti-tumor immune response, although these are preliminary findings and have yet to be thoroughly explored with immune-based evaluations.

The treatments applied in this study were larger and longer than any other published *in vivo* histotripsy ablations. For comparison, the #HOPE4LIVER clinical trial reported mean maximum liver tumor ablation dimensions (measured on post-treatment imaging and including a margin around the tumor) of 3.6 ± 1.4 cm and treatment durations of 34 ± 25 min^36^; this study reports mean maximum planned ablation dimensions of 4.5 ± 1.1 cm (no margin) and treatment durations of 111 ± 60 min. This largely pertains to the tumor sizes to be treated in these different studies; the dogs in this study had mean baseline tumor volumes of 107 ± 149 cm^3^, compared to 1.6 ± 2.0 cm^3^ in humans with liver cancer^36^. Similar to the #HOPE4LIVER trial, other clinical trials have performed multiple ablations of different lesions in a single patient, but never before have multiple treatments been performed in the same tumor over time. The fractionated treatments in this study were generally well-tolerated by the dogs in this study, and all dogs were discharged 1–3 hours post-treatment. Owners anecdotally reported that dogs tended to recover faster after subsequent treatments, with most dogs returning to their baseline level of pain, quality of life, and lameness by 1–3 days post-histotripsy. Any improvements in pain were then apparent after 3 days post-histotripsy. This corroborates with reports of thermal high-intensity FUS showing an onset of pain palliation at 3 days post-treatment in human bone metastases^18^.

Overall, histotripsy treatment did not show any significant changes in pain or quality of life. Notably, procedures were well tolerated and did now show any significant increases in pain or decreases in quality of life, which is promising for the future development of histotripsy for treating bone tumors. Generally, pain and quality of life varied per individual dog and their patient-specific treatment plan. For example, Patient #1 received a single treatment (9.8 cm^3^) in a relatively large tumor (129.1 cm^3^) and did not show any clinically significant improvements in pain. In contrast, Patient #2 received 2 treatments (23.9 cm^3^) in a slightly smaller tumor (97 cm^3^) and showed a clinically significant improvement in pain interference on the CBPI. 3 of the remaining 4 dogs with follow-up showed clinically significant improvements in pain severity. While this did not result in a statistically significant improvement in pain across all dogs, these findings are overall positive and warrant further investigation. Excitingly, histotripsy treatment also statistically significantly improved the pressure that dogs were putting on their tumor-bearing limb and had no significant negative effects on other gait analysis or lameness parameters. Increased pressure on gait analysis has been correlated to decreased pain in canines^37,38^. Together, these clinical outcomes suggest that histotripsy is a promising therapy for treating tumors while potentially reducing pain and improving gait in dogs with spontaneous bone tumors.

This clinical trial was focused on clinical outcomes of pain, quality of life, and gait. While survival was not an outcome measure in this study, we did record survival outcomes when possible. Future studies can be specifically designed to measure survival and metastatic progression. The survival ranged from 38 to 173 days post-treatment. For OS-bearing dogs without amputation and chemotherapy (pain medication only), survival is limited to 35 days^39^. Outside of the scope of this study, 2 patients in this trial elected for serial chest radiographs to evaluate for metastatic disease, showing a time to progression of 14 weeks (Patient #6) and 22 weeks (Patient #8). For dogs with OS undergoing amputation but no adjuvant chemotherapy, metastasis will typically present within 12 weeks^40^. It is interesting that our limited data on overall survival and time to metastatic progression are longer than the expected timelines for dogs with no definitive treatment, and future studies are needed to thoroughly evaluate time to progression and survival in histotripsy-treated bone tumor patients.

Interestingly, the patient that showed the longest time to progression eventually developed metastasis to the ribs. Canine and human OS metastasize primarily to the lungs, with spread to the bone being the second most common site of metastasis^41,42^. Promisingly, this patient showed only a 8.9% increase in whole tumor volume over 22 weeks, which is an atypically low amount for such an aggressive tumor if left untreated. This patient had the highest percent ablation of all dogs in this study, showing near complete ablation of the tumor volume (over 100% coverage of pre-treatment tumor size due to multiple overlapping treatments); the other dog with near complete ablation in this study, Patient #6, had the best improvement in lameness post-treatment, going from a lameness score of 5 (severe non-weight-bearing lameness) to a score of 0 (no detectable lameness). This suggests that targeting the majority of the tumor in future OS patients may yield the best results.

An important finding in this study was the occurrence of post-focal wounds in 4/24 treatments. These tended to occur more often in distal locations with little tissue (~ 1 cm) from the planned ablation to the post-focal aspect of the limb. We hypothesize that these wounds are thermal in nature and result from an accumulation of heat in the post-focal tissue of the extremities; in the typical treatment set-up, the pre-focal aspect of the limb is encased in degassed water, but the post-focal aspect is positioned on cotton cage pad covering a metal table. In more distal limb locations with less post-focal tissue, this could lead to a lack of tissue for heat dissipation and/or acoustic absorption during high PRF treatments. The incorporation of a post-focal circulating cooling water pad, along with lowering treatment parameters from 1500 Hz to 750 ppp to 1000 Hz and 500 ppp, led to 2 wounds out of 20 treatments in the second cohort of dogs (10%) as compared to 2 wounds out of 4 treatments in the first cohort treated (50%). Future studies are ongoing to evaluate this post-focal heating hypothesis, characterize factors that affect these post-focal wounds, and design improved strategies to mitigate thermal effects at high PRFs.

While the sample size of this study is limited and prohibits highly robust statistical analysis, the purpose of this trial was the pilot exploration of histotripsy in a limb-sparing setting for canine OS, focusing on safety and clinical outcomes. The appropriate statistical analyses were performed for this limited patient population, and future studies will build off of this first clinical trial to have larger datasets for increased statistical power to draw more meaningful conclusions. Additionally, a limitation of this study was the lack of immune data collection, as histotripsy has shown promising local and systemic immune effects that can contribute to anti-tumor immunity. This study was designed to evaluate the ablative and clinical effects of histotripsy ablation in canine OS; future treat-and-leave studies can incorporate blood sampling to evaluate systemic immune responses over time. In addition, future studies can consider combining histotripsy ablation with immunotherapy to further enhance the potential local and systemic histotripsy immune responses. This is especially pertinent in OS, where survival is limited by metastasis.

This first treat-and-leave canine clinical trial shows the safety, feasibility, and effectiveness of histotripsy for the ablation of large OS tumors, with promising long-term clinical outcomes. These findings can be explored and expanded further in future studies with a larger patient population and longer follow-up period. Future work in developing histotripsy for OS and other tumors will also need to focus on strategies for rapid ablation of large volumes, especially for mechanically stiffer targets such as OS that may require a higher dose for effective ablation. This can include increasing PRF combined with improved coupling strategies to reduce potential thermal effects, as well as considering alternative scanning strategies to maximize ablation efficiency. For OS specifically, the ability to deliver non-uniform dosing to bone versus soft tissue portions of tumor could reduce the amount of time needed to ablate the whole tumor by lowering the dose for soft tissue regions. Additionally, varying amounts of bone versus soft tissue components may affect clinical outcomes, which could be explored further in future studies. Lastly, the long-term immunomodulatory effects of histotripsy should be explored further in the context of OS, including combinations of histotripsy with immunotherapy to enhance the potential anti-tumor immune response. Overall, this work is a promising step forward for the clinical translation of histotripsy as a non-invasive limb-sparing therapy for dogs and humans with OS.

## Supplementary Information

Below is the link to the electronic supplementary material.

Supplementary Material 1

Supplementary Material 2

Supplementary Material 3

## Data Availability

The data presented in this study are available within the main manuscript and supplementary information files. Any additional data not explicitly detailed in the manuscript or supplementary files are available upon reasonable request by reaching out to the corresponding author.
