# Supplementary material for: The first limb-sparing use of histotripsy for canine osteosarcoma
Source: Sci Rep. 2026 Mar 23;16:14574. doi: 10.1038/s41598-026-42319-z (PMC13153271; doi:10.1038/s41598-026-42319-z)
Supplement: Supplementary file 1 — Supplementary Material 1 [file 41598_2026_42319_MOESM1_ESM.docx]

The First Treat-and-Leave Clinical Trial of Histotripsy for Canine Osteosarcoma: Clinical Outcomes After Fractionated Treatments

**Supplementary Figures**

**
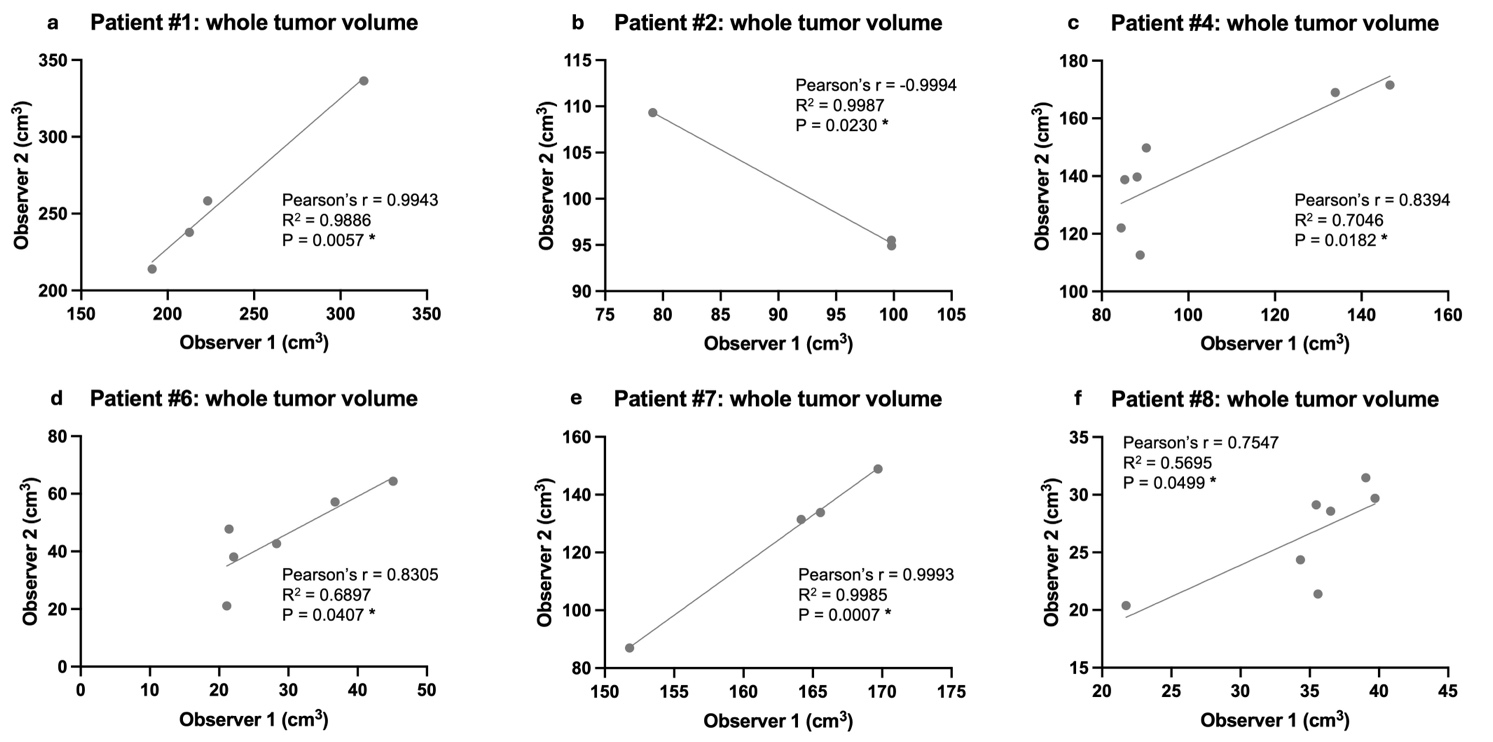
**

**Supplementary Figure S1.** Whole tumor segmentations correlated between two observers for each canine OS patient. Pearson’s correlations were used to compare tumor volumes between Observer 1 (E.R.V.) and Observer 2 (J.S.K.). All correlations are significant (P < 0.05).

**Supplementary Figure S2.** Videos of a representative canine OS clinical trial patient (#6) walking before and 1 month after histotripsy treatment(s), showing a marked decrease in lameness after treatment.

**Supplementary Figure S3.** Videos of a representative canine OS clinical trial patient (#7) walking before and 1 month after histotripsy treatment(s), showing a mild decrease in lameness after treatment.

**
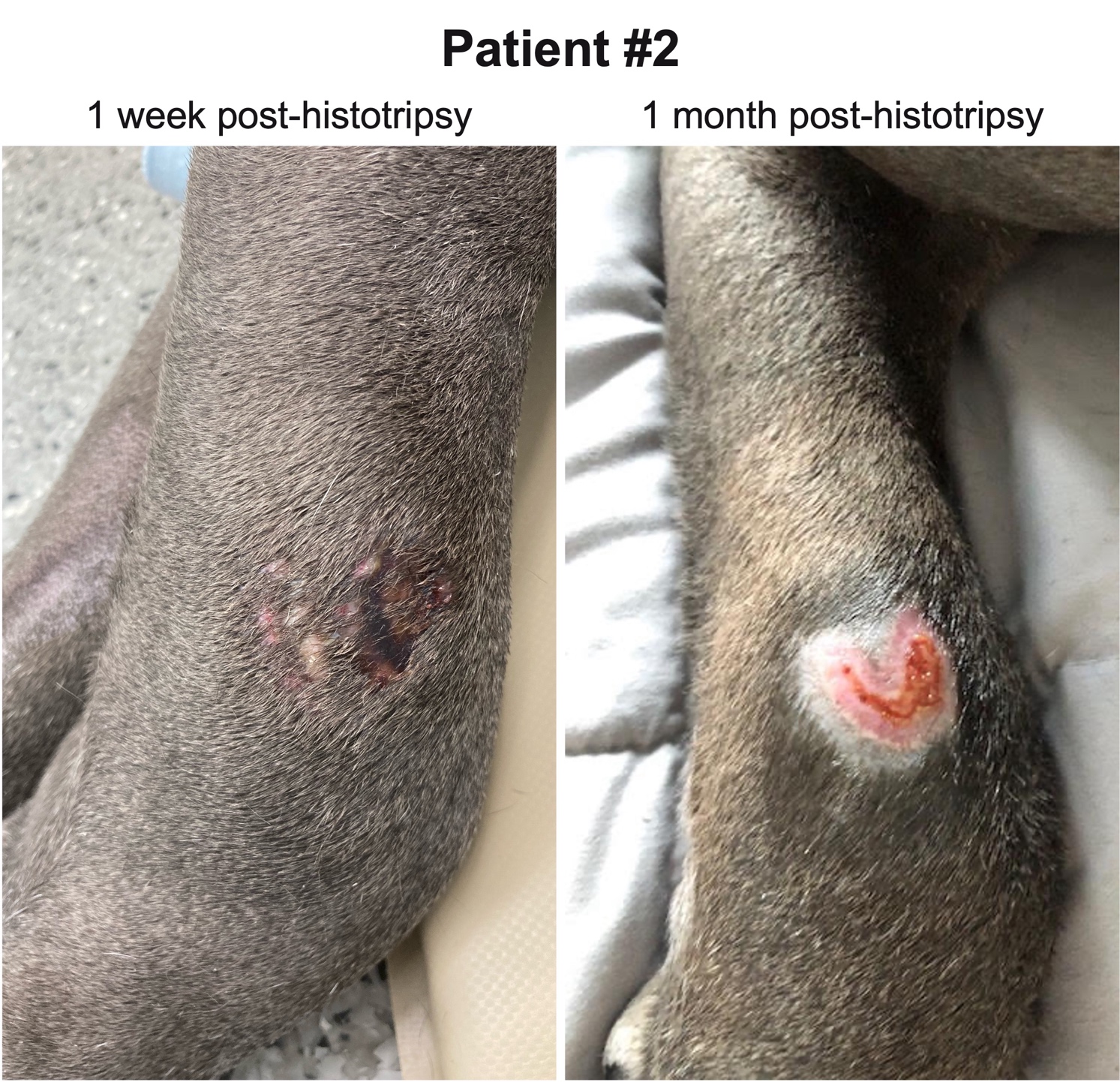
**

**Supplementary Figure S4.** Post-focal wound after the second histotripsy treatment in a 5-year-old male neutered Great Dane (Patient #2).

**Supplementary Tables**

**Supplemental Table S1.** Full histotripsy treatment parameters and characteristics for each individual patient treatment in the present study.

| **Patient** | **Treatment** | **Treatment**  **Volume (cm^3^)** | **Treatment**  **Pressure (MPa)** | **Treatment**  **Depth (cm)** | **Visible Bubble**  **Cloud?** | **Pre-Focal**  **Cavitation?** | **Adverse**  **Event(s)** |
| --- | --- | --- | --- | --- | --- | --- | --- |
| 1 | 1 | 9.8 | -33.2 | 3.1 | Yes | No | None |
| 2 | 1 | 9.8 | -42.1 | 2.4 | No | Moderate on skin & bone | None |
|  | 2 | 14.1 | -53.7 | 2.7 | No | Mild on skin & bone | Skin ulceration grade 1 |
| 3 | 1 | 4.7 | -39.5 | 1.7 | No | Moderate on skin | Skin ulceration grade 3 |
| 4 | 1 | 19.6 | -45.7 | 2.5 | Intermittent, in bone | Moderate on skin & bone | None |
|  | 2 | 18 | -41.2 | 2 | Intermittent, in soft tissue | Mild on skin | None |
|  | 3 | 16.4 | -43.9 | 2.1 | Intermittent, in soft tissue | Mild on skin | None |
|  | 4 | 8.0 | -44.8 | 2.4 | No | Moderate on skin | None |
| 5 | 1 | 29.2 | -34.1 | 3.2 | Yes | No | None |
| 6 | 1 | 7.9 | -43.9 | 1.8 | No | Moderate on skin | None |
|  | 2 | 1.6 | -42.1 | 1.5 | Yes | No | None |
|  | 3 | 4.1 | -36.8 | 1.3 | No | Mild on skin | None |
|  | 4 | 17.7 | -42.1 | 1.9 | Intermittent, in soft tissue | Moderate on skin | None |
|  | 5 | 5.3 | -44.8 | 1.8 | No | Mild on skin | Skin ulceration grade 3 |
| **7** | 1 | 26.2 | -49.2 | 3.2 | No | Mild on skin | None |
|  | 2 | 26.2 | -50.1 | 3.5 | Intermittent, in soft tissue | Mild on skin | None |
|  | 3 | 17.3 | -47.5 | 2.2 | No | No | None |
|  | 4 | 4.1 | -39.5 | 1.8 | Intermittent, in soft tissue | Mild on skin | None |
|  | 5 | 18.9 | -47.5 | 3.4 | Intermittent, in bone | No | None |
| 8 | 1 | 15.7 | -40.4 | 2.2 | Intermittent, in bone | Moderate on skin | None |
|  | 2 | 11.8 | -41.2 | 1.4 | Intermittent, in soft tissue | Moderate on skin | None |
|  | 3 | 6.3 | -33.2 | 1.6 | Intermittent, in soft tissue | Moderate on skin | None |
|  | 4 | 8.2 | -46.6 | 2.2 | Intermittent, in bone | Mild on skin | None |
| 9 | 1 | 19.6 | -45.7 | 2.1 | Intermittent, in bone | Moderate on skin | Erythema grade 1 |
| **Mean** $\boldsymbol{\pm}$  **Standard Deviation** | | **13.4** $\boldsymbol{\pm}$ **7.7** | **-42.9** $\boldsymbol{\pm}$ **5.3** | **2.3** $\boldsymbol{\pm}$ **0.6** | **Yes: 2/24**  **Intermittent: 12/24**  **No: 9/24** | **Moderate: 10/24**  **Mild: 9/24**  **No: 5/24** | **No: 20/24**  **Yes: 4/24** |

**Supplemental Table S2.** Gait analysis parameters for all 6 dogs with follow-up data in the present study at the start and end of each dog’s follow-up periods.

| **Patient** | **Start & End of Follow-Up Period** | **Pressure-Plate Walkway Assessment** | | | | **Veterinarian Assessment** |
| --- | --- | --- | --- | --- | --- | --- |
|  |  | **Peak Pressure**  (kPa) | **Stance Time**  (sec) | **Body Weight**  **Distribution** (%) | **Vertical Impulse**  **Distribution** (%) | **Lameness Score**  (1-5) |
| 1 | Start | 125.40 | 0.38 | 31.52 | 36.86 | 1.0 |
|  | End | 143.25 | 0.29 | 30.09 | 34.29 | 2.0 |
| 2 | Start | 122.40 | 0.47 | 16.12 | 16.57 | 2.0 |
|  | End | **232.40 +** | 0.51 | **32.80 +** | **32.36 +** | 0.5 |
| 3 | Start | 47.00 | 0.19 | 4.15 | 1.40 | 3.0 |
|  | End | 98.50 | 0.25 | 5.35 | 2.26 | 3.5 |
| 4 | Start | 110.40 | 0.40 | 15.84 | 15.34 | 3.0 |
|  | End | 146.40 | 0.40 | 19.44 | 19.34 | 0.0 |
| 5 | Start | 87.00 | 0.47 | 9.70 | 6.70 | 2.0 |
|  | End | 97.50 | 0.43 | 9.65 | 6.30 | 3.0 |
| 6 | Start | 103.00 | 0.31 | 17.92 | 16.12 | 2.0 |
|  | End | 106.33 | 0.26 | 20.62 | 14.80 | 2.5 |
| **Mean** $\boldsymbol{\pm}$  **Standard Deviation** | Start | 102.2 $\pm$ 32.7 | 0.4 $\pm$ 0.1 | 15.9 $\pm$ 9.2 | 15.5 $\pm$ 12.1 | 2.2 $\pm$ 0.8 |
|  | End | 137.4 $\pm$ 51.4 | 0.4 $\pm$ 0.1 | 19.7 $\pm$ 10.8 | 18.2 $\pm$ 13.2 | 1.9 $\pm$ 1.4 |
|  | P value | **0.04 *** | 0.59 (ns) | 0.22 (ns) | 0.37 (ns) | 0.72 (ns) |

+ indicates statistical significance (P < 0.05) for an individual dog’s walkway replicates as determined by unpaired t tests. * indicates statistical significance (P < 0.05) across all dogs as determined by paired t tests or non-parametric equivalent.

**Supplemental Table S3.** Pain and quality of life scores at the start and end of each dog’s follow-up periods for all 6 dogs with follow-up data in the present study.

| **Patient** | **Start & End of Follow-Up Period** | **Canine Owner-Reported Quality of Life (CORQ)** 1-10 | | | | **Canine Brief Pain Inventory (CBPI)** 0-7 | |
| --- | --- | --- | --- | --- | --- | --- | --- |
|  |  | **Vitality** | **Companionship** | **Mobility** | **Overall Pain** | **Pain Severity** | **Pain Interference** |
| 1 | Start | 5.6 | 7.0 | 7.0 | 0.0 | 0.5 | 0.0 |
|  | End | 4.8 | 6.7 | 6.5 | 3.5 | 1.3 | 1.2 |
| 2 | Start | 3.6 | 6.2 | 2.5 | 4.5 | 5.0 | 6.8 |
|  | End | 3.8 | 6.5 | 4.8 | 2.0 | 4.5 | **3.7 *** |
| 3 | Start | 4.2 | 6.5 | 2.3 | 4.5 | 4.5 | 5.3 |
|  | End | 3.8 | 6.7 | 3.5 | 2.0 | **3.0 *** | 4.3 |
| 4 | Start | 5.2 | 6.0 | 6.5 | 3.0 | 5.0 | 0.8 |
|  | End | 5.0 | 6.0 | 5.8 | 1.5 | **3.3 *** | 1.2 |
| 5 | Start | 3.2 | 6.3 | 3.0 | 1.0 | 2.8 | 3.2 |
|  | End | 2.8 | 6.4 | 3.5 | 2.0 | **1.8 *** | 6.2 |
| 6 | Start | 3.6 | 6.2 | 4.8 | 2.5 | 1.3 | 2 |
|  | End | 5.6 | 6.8 | 6.8 | 0.5 | 0.6 | 1.8 |
| **Mean** $\boldsymbol{\pm}$  **Standard Deviation** | Start | 4.2 $\pm$ 1.0 | 6.4 $\pm$ 0.4 | 4.4 $\pm$ 2.1 | 2.6 $\pm$ 1.8 | 3.2 $\pm$ 2.0 | 3.0 $\pm$ 2.6 |
|  | End | 4.3 $\pm$ 1.0 | 6.5 $\pm$ 0.3 | 5.2 $\pm$ 1.5 | 1.9 $\pm$ 1.0 | 2.4 $\pm$ 1.4 | 3.0 $\pm$ 2.0 |
|  | P value | 0.41 (ns) | 0.28 (ns) | 0.18 (ns) | 0.53 (ns) | 0.08 (ns) | 0.95 (ns) |

* indicates clinically significant reduction in pain based on CBPI guidelines ($\geq$1 reduction in pain severity, $\geq$2 reduction in pain interference).
